# Automated Digital Quantification of Pulmonary Fibrosis in Human Histopathology Specimens

**DOI:** 10.3389/fmed.2021.607720

**Published:** 2021-06-15

**Authors:** Lauren C. Testa, Yvon Jule, Linnea Lundh, Karine Bertotti, Melissa A. Merideth, Kevin J. O'Brien, Steven D. Nathan, Drew C. Venuto, Souheil El-Chemaly, May Christine V. Malicdan, Bernadette R. Gochuico

**Affiliations:** ^1^Medical Genetics Branch, National Human Genome Research Institute, National Institutes of Health, Bethesda, MD, United States; ^2^Biocellvia, Marseille, France; ^3^Office of the Clinical Director, National Human Genome Research Institute, National Institutes of Health, Bethesda, MD, United States; ^4^Advanced Lung Disease and Lung Transplant Program, Inova Fairfax Hospital, Falls Church, VA, United States; ^5^Division of Pulmonary and Critical Care Medicine, Brigham and Women's Hospital, Boston, MA, United States; ^6^Undiagnosed Diseases Program, Office of the Director, National Institutes of Health, Bethesda, MD, United States

**Keywords:** Ashcroft score, idiopathic pulmonary fibrosis, Hermansky-Pudlak syndrome, collagen, interstitial lung disease

## Abstract

Pulmonary fibrosis is characterized by abnormal interstitial extracellular matrix and cellular accumulations. Methods quantifying fibrosis severity in lung histopathology samples are semi-quantitative, subjective, and analyze only portions of sections. We sought to determine whether automated computerized imaging analysis shown to continuously measure fibrosis in mice could also be applied in human samples. A pilot study was conducted to analyze a small number of specimens from patients with Hermansky-Pudlak syndrome pulmonary fibrosis (HPSPF) or idiopathic pulmonary fibrosis (IPF). Digital images of entire lung histological serial sections stained with picrosirius red and alcian blue or anti-CD68 antibody were analyzed using dedicated software to automatically quantify fibrosis, collagen, and macrophage content. Automated fibrosis quantification based on parenchymal tissue density and fibrosis score measurements was compared to pulmonary function values or Ashcroft score. Automated fibrosis quantification of HPSPF lung explants was significantly higher than that of IPF lung explants or biopsies and was also significantly higher in IPF lung explants than in IPF biopsies. A high correlation coefficient was found between some automated quantification measurements and lung function values for the three sample groups. Automated quantification of collagen content in lung sections used for digital image analyses was similar in the three groups. CD68 immunolabeled cell measurements were significantly higher in HPSPF explants than in IPF biopsies. In conclusion, computerized image analysis provides access to accurate, reader-independent pulmonary fibrosis quantification in human histopathology samples. Fibrosis, collagen content, and immunostained cells can be automatically and individually quantified from serial sections. Robust automated digital image analysis of human lung samples enhances the available tools to quantify and study fibrotic lung disease.

## Introduction

Pulmonary fibrosis is a chronic interstitial lung disease (ILD) characterized by alveolar parenchymal accumulation of extracellular matrix protein, mesenchymal cells, and immune cells that disrupts normal lung architecture (1). Although the etiology of pulmonary fibrosis has not been fully elucidated, some mechanisms contributing to the pathobiology of disease have been identified. Several disorders are known to be associated with lung fibrosis, including idiopathic pulmonary fibrosis (IPF), collagen vascular disorders, telomere disease, Erdheim-Chester disease, and Hermansky-Pudlak syndrome (HPS).

IPF, a prototypical fibrotic lung disease, typically develops in older individuals. The incidence of IPF is ~2.8–9.3 per 100,000 per year in North America and Europe, and the prevalence is 10–60 cases per 100,000 people (1, 2). In patients older than 65, the prevalence increases to 400 per 100,000 people (2). Patients with IPF present with insidious onset of dyspnea on exertion and chronic non-productive cough. Computed tomography (CT) scans show bilateral interstitial reticulations, traction bronchiectasis, and honeycombing with subpleural predominance. Pirfenidone and nintedanib are anti-fibrotic drugs approved by the Food and Drug Administration (FDA) as treatment for IPF, and lung transplantation may be performed in suitable candidates with severe disease (3, 4). IPF is associated with a poor prognosis. Estimated survival of untreated patients is ~3–5 years after onset of symptoms, but treatment with pirfenidone was shown to improve survival (2, 5).

HPS is an autosomal recessive disorder characterized by improper biogenesis of lysosome-related organelles (6). Locus heterogeneity is a feature of HPS, and eleven genetic types of HPS are reported (6, 7). HPS is a rare disorder, affecting 1–9 per 1,000,000 people (8). However, the prevalence of HPS-1 is higher in Puerto Rico due to a genetic founder effect in the northwest area of the island, where ~1 in 1,800 people are affected by HPS-1 (9). Patients with HPS-1 from northwest Puerto Rico are typically homozygous for a 16-base pair duplication in *HPS1* (c.1472_1487dup16) (10). All patients with HPS manifest with oculocutaneous albinism and a bleeding diathesis to some degree, and granulomatous colitis can develop in a subpopulation of patients (8). Other clinical stigmata of disease are dependent upon HPS genetic type, including neutropenia (HPS-2), immunodeficiency (HPS-2), and pulmonary fibrosis (HPS-1, HPS-2, HPS-4, and possibly HPS-10) (8, 11). Pulmonary fibrosis is highly prevalent in patients with HPS-1 regardless of their *HPS1* genetic variants (12). Nearly all adults with HPS-1 will develop fibrotic lung disease with most presenting with symptomatic pulmonary fibrosis in their middle ages (13). High-resolution CT scans findings are similar to those in IPF, except ground glass opacification can be found more frequently in CT scans of patients with HPS pulmonary fibrosis (14, 15). There are no FDA-approved treatments for HPS pulmonary fibrosis (16–18). Lung transplantation is a viable option for some patients with end-stage HPS pulmonary fibrosis (19).

Research is indicated to improve the understanding of these devastating diseases, and estimation of severity of pulmonary fibrosis is useful for studying this disorder. Clinical severity of disease is generally estimated by lung physiology tests and radiology findings (2). Methods to quantify severity of disease in fibrotic lung histopathology samples are limited. Initially reported in 1988, Ashcroft scoring of lung tissue continues to be utilized as a method to quantify severity of pulmonary fibrosis in stained histologic sections (20). Ashcroft scoring utilizes a numerical scale from 0 through 8 to grade fibrosis; it is subject to intra- and inter-scorer variability (20). Computerized digital image analysis was developed to utilize a continuous measure of fibrosis in various rodent or human tissues, including lung, liver, heart, and kidney (21–28). Most computerized digital image analyses evaluate severity of fibrosis by quantifying collagen content stained by picrosirius red or to a lesser extent by Masson trichrome or immunostaining. The quantification of collagen is assessed from the ratio of the stained collagen area vs. the area of analyzed regions of interest selected in the whole section. Histological scoring and digital image analysis involve assessment of several random fields per section, and systematic analysis of the entire tissue section is generally not performed due to feasibility issues. Lung tissue is heterogeneously affected by pulmonary fibrosis whereby fibrotic and less affected or normal appearing tissue localize in adjacent regions (14). Thus, it is possible that assessment of fibrosis from random fields may inadvertently overestimate or underestimate the degree of fibrosis in the entire lung section.

Given these limitations, there is a need to develop a quantitative, accurate, rapid, and reader-independent assessment of pulmonary fibrosis severity in entire tissue sections. Previously, we developed an automated software program which meets these criteria using a murine model of bleomycin-induced pulmonary fibrosis (29). In this pilot study, we explored whether this software program could quantify fibrosis in a small number of human lung tissue specimens obtained from HPS pulmonary fibrosis and IPF patients and biopsies from IPF patients. Digital quantitative analyses of fibrosis, collagen content, and CD68 immunolabeling, a marker of inflammation, were performed from the same or serial histological sections. To compare and validate the severity of pulmonary fibrosis using our digital analysis, correlation was established with our quantitative measurements and lung function values. Overall, we show that this digital histological analysis of pulmonary fibrosis can be readily applied to human samples, and further studies with a larger sample size are indicated. This tool provides robust, accurate, reader-independent quantification of pulmonary fibrosis and enhances the available methods to study fibrotic lung disease.

## Materials and Methods

### Patient Selection

Patients provided written informed consent and enrolled in protocol 04-HG-0211 (Clinical Trials number, NCT00084305; Procurement and Analysis of Specimens from Individuals with Pulmonary Fibrosis), which was approved by the institutional review board of the National Human Genome Research Institute. IPF was diagnosed in accordance with ATS/ERS/JRS/ALAT guidelines (30). HPS was diagnosed in patients with clinical manifestations of disease (e.g., oculocutaneous albinism, bleeding diathesis) who had absent platelet dense granules on whole mount electron microscopy examination as described (31, 32). Pulmonary fibrosis was detected by characteristic findings on high-resolution CT scan of the chest (13, 14). Variants in *HPS1* were identified by polymerase chain reaction and gel electrophoresis, Sanger sequencing, or whole exome sequencing as described (16, 33).

### Chest Radiographic Imaging and Pulmonary Function Testing

High-resolution CT scans of the chest were performed without intravenous contrast during end-inspiration in the prone position as described (34). Pulmonary function measurements, including forced vital capacity (FVC), forced expiratory volume in 1-second (FEV_1_), total lung capacity (TLC), and diffusion capacity adjusted for hemoglobin (DLCO_adj_), were performed in accordance with American Thoracic Society/European Respiratory Society standards as described (35).

### Lung Tissue Staining

Lung explant and biopsy samples were obtained from clinically-indicated procedures as described (36). Explanted specimens from three right lung lobes were obtained from three patients with HPS pulmonary fibrosis and three patients with IPF. Biopsies from two right lung lobes were obtained from four patients with IPF. Samples were fixed in 10% neutral buffered formalin, embedded in paraffin, and sectioned with a microtome. Serial lung sections 5 μm in thickness were stained with (i) hematoxylin and eosin to assess general morphology and to perform Ashcroft scoring and (ii) picrosirius red and alcian blue for quantification of collagen fibers and mucus.

### Automated Histological Quantification and Ashcroft Scoring

Lung slices, which could include more than one section, were scanned at 20 × magnification using a NanoZoomer-SQ (Hamamatsu, Japan) and digital images of whole lung sections were captured (pixel size: 0.452 μm) using NDP.view 2 Hamamatsu software. Entire sections stained with picrosirius red and alcian blue [HPS pulmonary fibrosis explants [three patients, 10 sections], IPF explants [three patients, nine sections], IPF biopsies [4 patients, 23 sections]] were analyzed using image processing software patented by Biocellvia (Biocellvia, France). Alcian blue staining was added to picrosirius red to stain mucus in alveolar spaces and makes it possible to discriminate and exclude mucus contained in alveoli. As previously described, quantification of fibrosis using this Biocellvia proprietary software program was assessed on the basis of pulmonary fibrosis tissue density. For each microtile, density value corresponds to the area of stained area vs. microtile area ranging from 0 to 1. Three morphometric parameters were performed on the same digital lung image from picrosirius red staining: (i) mean pulmonary tissue density, (ii) fibrosis score (referred to as pulmonary foci in reference 29), and (iii) collagen content (29). Using this software program, mean pulmonary tissue density and fibrosis score were determined from digital images in which RGB values of each pixel were converted to a density value, ranging between 0 to 1, according to tissue density. The absence of parenchymal tissue (e.g., airspace, vessel lumen) is characterized by a value of 0, and the presence of parenchymal tissue by continuous values up to 1. Mean pulmonary tissue density corresponds to the mean value of whole pixel density values distributed in the entire lung section area. Mean fibrosis score of a lung section corresponds to the percentage of high individual tissue density values (i.e., >0.75) which are specifically expressed in fibrotic areas (29). Percentage of collagen content was calculated, simultaneously and automatically from the same lung sections used to assess fibrosis, as the ratio of the area of picrosirius red stained collagen fibers to the entire lung section area. Data were automatically generated by the software program and were independent of the operator.

Fibrosis severity was also evaluated on serial hematoxylin and eosin stained sections by three scorers (LCT, LL, BRG) blinded to patient group according to the scale defined by Ashcroft (20) from digital images of stained sections viewed at 200 × magnification. Ten randomly chosen fields from each section were scored. Fields with large blood vessels and airways were excluded, and adjacent lung parenchyma was analyzed. Mean Ashcroft score was recorded from HPS explants (10 sections from three patients), IPF explants (eight sections from three patients), and IPF biopsies (20 sections from four patients).

### Immunohistochemistry

Immunostaining for CD68 was performed on slides treated with hydrogen peroxide and methanol to quench endogenous peroxidase activity. Antigen retrieval was performed using Tris/EDTA buffer (pH 8.0). After rinsing with deionized water and incubation in TBS (20 min, room temperature), lung sections were incubated with rabbit anti-CD68 antibody (ab213363, dilution 1:7,000, Abcam, France) for 1 h at room temperature. Slides were rinsed with TBS and incubated with HRP anti-rabbit IgG (ImmPRESS HRP, MP-7401, Vector Laboratories, United Kingdom) for 45 min at room temperature. Hematoxylin counter stain was applied. Percentage of CD68 staining was calculated as the ratio of the area of CD68 stained cells to the entire lung section area.

### Statistical Analysis

Data are presented as mean ± standard error of mean. Statistical differences between groups were analyzed by two-tailed Student's t-tests. Spearman correlation coefficient was calculated to correlate automated density or fibrosis scores with pulmonary function measurements or Ashcroft values. A *p* < 0.05 was considered statistically significant. Two-factor ANOVA without replication was performed based on the agreement in Ashcroft scores between raters and was used to calculate the intraclass correlation coefficient (37). An intraclass correlation coefficient from 0.75 to 0.90 indicates “good” reliability (38).

## Results

### Patient Characteristics

**Figure 1:**
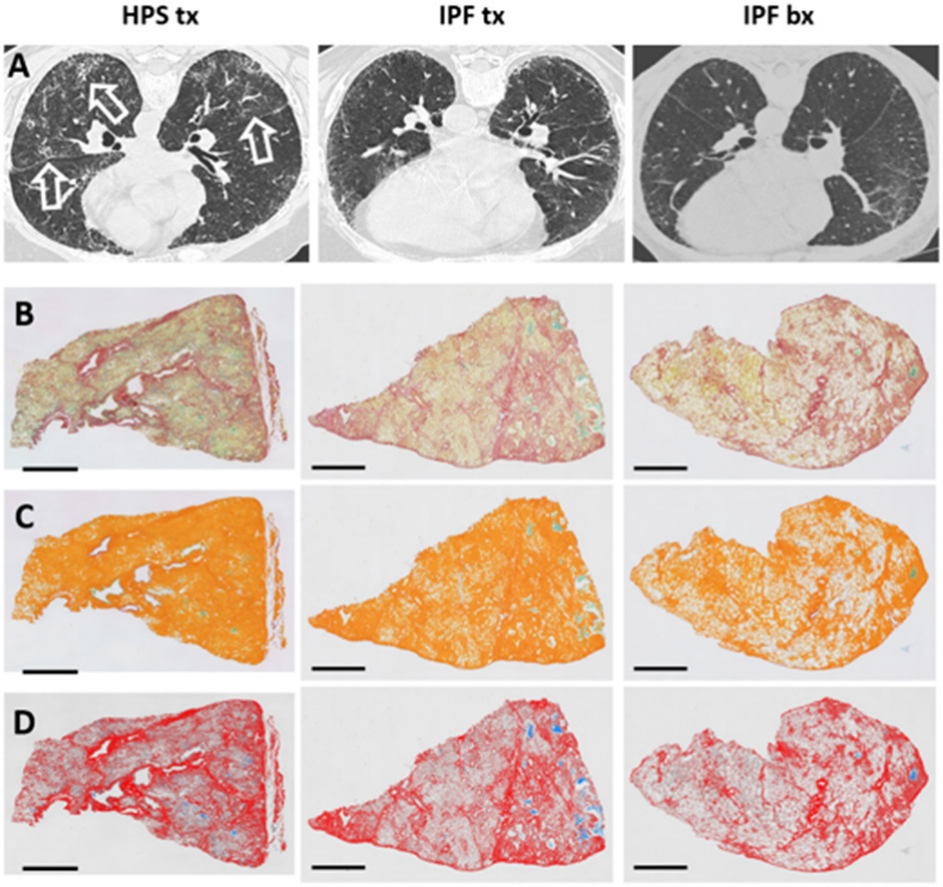
High-resolution Chest CT Scan and Histology Images of Patients with Pulmonary Fibrosis. Representative high-resolution chest CT scan images **(A)** show reticulations and honeycombing that are more severe in Hermansky-Pudlak syndrome (HPS) pulmonary fibrosis lung explant (HPS tx, left column) and idiopathic pulmonary fibrosis (IPF) lung explant (IPF tx, middle column) tissue donors than in IPF patients who had lung biopsies (IPF bx, right column). High-resolution CT scans from HPS pulmonary fibrosis patients also demonstrate ground glass opacifications (open arrows). Native images of lung sections stained with picrosirius red and alcian blue **(B)** shows collagen fibers (red), parenchymal tissue (yellow), and mucus (blue-green). Areas of pulmonary fibrosis (orange pseudocolor) are quantified, and mucus (green) is excluded from quantification of fibrosis **(C)**. Parenchymal tissue (pseudocolored in gray) and mucus (stained with alcian blue) are not considered in the quantification of collagen (row **D**). Size bar = 2 mm.

Lung tissue specimens fixed in formalin were obtained from three patients with HPS pulmonary fibrosis and seven patients with IPF. Mean age of explanted lung tissue donors with HPS pulmonary fibrosis was 54 ± 5.7 years, which did not differ significantly from that of IPF explant or IPF biopsy tissue donors, whose ages were 64 ± 0.83 and 64 ± 2.9 years, respectively (Table 1). High-resolution CT scan images from the patients with HPS pulmonary fibrosis or IPF showed interstitial reticulations and honeycombing, and only images from the patients with HPS pulmonary fibrosis contained regions with ground glass opacification (Figure 1). Mean pulmonary function test values in patients with HPS pulmonary fibrosis or IPF explant donors were lower than those of IPF biopsy donors; TLC and DLCO_adj_ were significantly lower in HPS pulmonary fibrosis (*p* = 0.037 and *p* = 0.018, respectively) or IPF explant donors (*p* = 0.020 and *p* = 0.048, respectively) than in IPF biopsy donors (Table 1).

**Table 1:** Patient characteristics.

	**HPS tx (*n* = 3)**	**IPF tx (*n* = 3)**	**IPF bx (*n* = 4)**	***p*-value (HPS tx v IPF tx, HPS tx v IPF bx, IPF tx v IPF bx)**
Age (years)	54 ± 5.7	64 ± 0.83	64 ± 2.9	0.161 0.148 0.980
Gender (male/female)	1/2	2/1	3/0	
FVC (% predicted)	48 ± 7.0	47 ± 2.1	76 ± 9.0	0.898 0.071 0.044
FEV1 (% predicted)	51 ± 7.8	63 ± 2.4	96 ± 12	0.206 0.036 0.075
TLC (% predicted)	42 ± 9.2	48 ± 1.2	72 ± 5.9	0.574 0.037 0.020
DLCO (% predicted)	24 ± 5.6	37 ± 1.3	67 ± 9.8	0.085 0.018 0.048

*HPS, Hermansky-Pudlak syndrome; IPF, idiopathic pulmonary fibrosis; tx, transplant; bx, biopsy; FVC, forced vital capacity; FEV1, forced expiratory volume in 1 s; TLC, total lung capacity; DLCO, diffusion capacity*.

### Automated Quantification of Pulmonary Fibrosis

**Figure 2:**
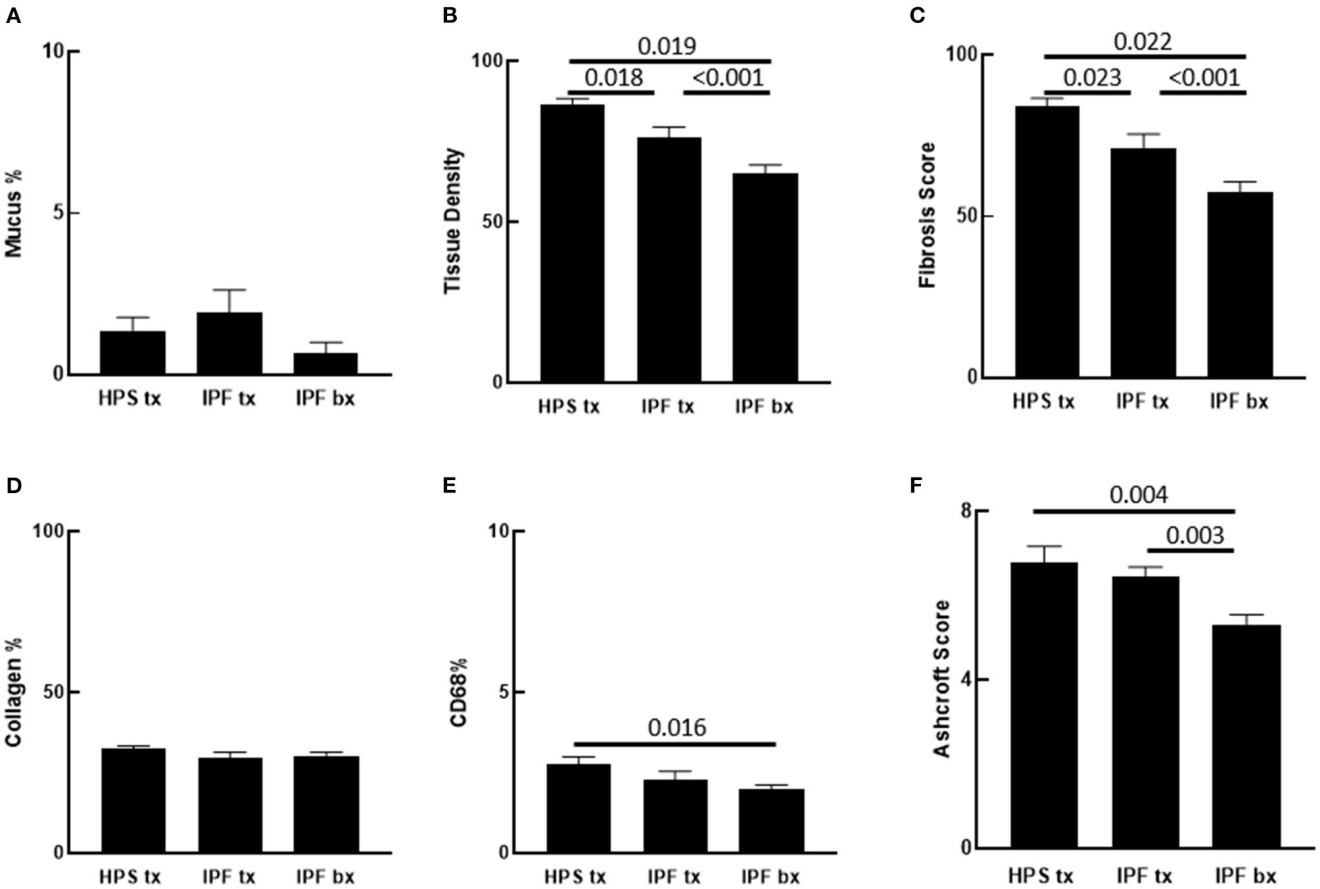
Quantification of Pulmonary Fibrosis and CD68 Immunostaining. Automated quantification of pulmonary fibrosis was performed on tissue sections from Hermansky-Pudlak syndrome lung explant (HPS tx, *n* = 10), idiopathic pulmonary fibrosis lung explant (IPF tx, *n* = 9), and idiopathic pulmonary fibrosis lung biopsy (IPF bx, *n* = 23) specimens stained with picrosirius red and alcian blue. **(A)** Mucus percentage, defined as the ratio of alcian blue stained mucus area divided by the total section area, did not differ significantly between the three groups. **(B,C)** Pulmonary tissue density and fibrotic score percentage were significantly higher in HPS pulmonary fibrosis and IPF explant sections than in IPF biopsy sections. Pulmonary tissue density and fibrotic score percentage were also significantly higher in HPS pulmonary fibrosis explant sections than in IPF explant sections. **(D)** Percentages of collagen content did not differ significantly between sections from the three groups. **(E)** Percentages of CD68 staining was significantly higher in HPS pulmonary fibrosis explant sections than in IPF biopsy sections. **(F)** Mean Ashcroft scores were significantly higher in HPS pulmonary fibrosis and IPF explant sections than in IPF biopsy sections.

Tissue fibrosis was quantified in whole lung sections from HPS pulmonary fibrosis explants, IPF explants, and IPF biopsies stained with picrosirius red (Figure 1). A technical challenge that needed to be overcome prior to lung section analysis was the presence of mucus within the airspace areas Accumulation of mucus could not be discriminated from picrosirius red staining alone and inadvertently contributed to the assessment of parenchymal tissue density and fibrosis scores. To address this issue, alcian blue staining was added to picrosirius red to specifically discriminate mucus and be excluded automatically from airspace area by means of dedicated algorithms (Figures 1, 2). We found no statistically significant differences in mucus percentage between the three groups (Figure 2A).

Analysis of parenchymal tissue density revealed that HPS explants had significantly higher mean tissue density than IPF explants (*p* = 0.018) and IPF biopsies (*p* = 0.019). Likewise, density of IPF explants was significantly higher than that of IPF biopsies (*p* < 0.001) (Figure 2B). Consistent with these data, fibrosis score of HPS explant tissues was significantly higher than that of IPF explant tissues (*p* = 0.023) and IPF biopsies (*p* < 0.001), and fibrosis score of IPF explant tissues was significantly higher than that of IPF biopsy tissues (*p* = 0.022) (Figure 2C).

To determine collagen expression in the alveolar interstitial extracellular matrix and its contribution to automated tissue density and fibrosis values, lung tissue collagen content was quantified simultaneously in the same picrosirius red stained lung sections that were analyzed for fibrosis using our software. No statistically significant differences were found in collagen content between lung specimens from the three patient groups. Notably, the Biocellvia imaging analysis software for the measurement of tissue density and fibrosis score parameters as well as collagen content was reproducible, because we found that repeated analyses of at least 20% of digital images had a standard error of 0 ± 0 pixels (data not shown). Overall, these results suggest that factors other than collagen content contribute to high automated tissue density values and fibrosis scores in HPS explanted tissue (Figure 2D).

**Figure 3:**
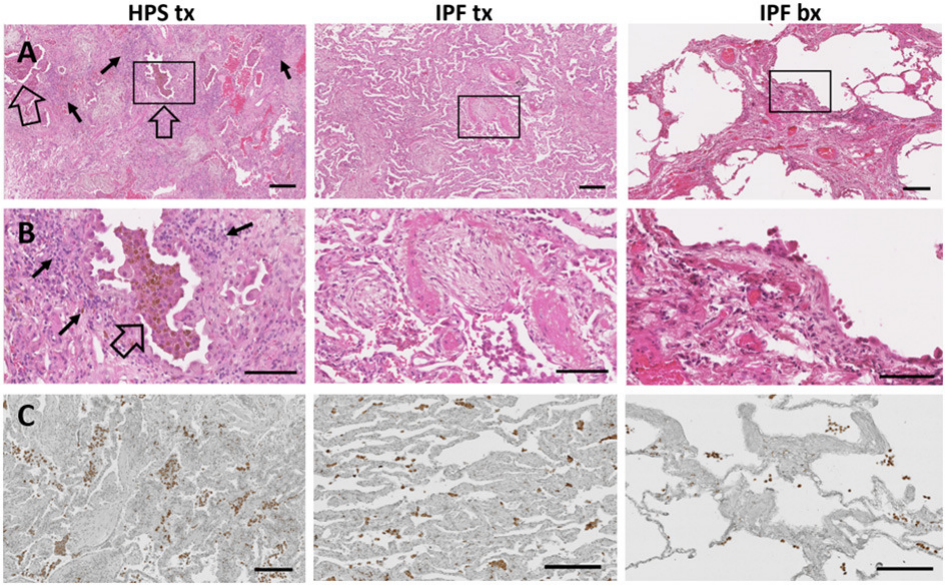
Immune Cells in Pulmonary Fibrosis Tissue. Representative low magnification **(A)** and high magnification **(B)** fields of hematoxylin and eosin stained lung tissue from HPS explant (HPS tx, left column), IPF explant (IPF tx, middle column), and IPF biopsy (IPF bx, right column) are shown. Severe fibrosis is found in HPS pulmonary fibrosis explant and IPF explant specimens; moderate fibrosis is demonstrated in IPF biopsy samples. Aggregates of alveolar macrophages (open arrow) and interstitial inflammatory cells (solid arrow) are observed in HPS transplant cases. Immunostaining with primary anti-CD68 antibody **(C)** shows aggregates of macrophages (brown) in the alveolar spaces and interstitium of pulmonary fibrosis specimens. CD68-labeled cells appear more prominent in HPS pulmonary fibrosis explant sections than in IPF explant or biopsy sections. Size bar = 200 um **(A)**, = 100 um **(B)**, or = 190 um **(C)**.

In addition to fibrosis, cellular infiltrates are present within the lung parenchyma in HPS pulmonary fibrosis. To develop digital quantification of inflammation associated with pulmonary fibrosis, we immunostained macrophages in tissue sections. This allowed us to develop algorithms specific to the automatic digital quantification of immunolabelled cells that can be used later for more in-depth analyses of lung inflammation. We previously reported high concentrations of alveolar macrophages in bronchoalveolar lavage fluid from patients with HPS (39). We then sought to determine whether increased macrophages in the alveolar interstitium contribute to high fibrosis score in HPS pulmonary fibrosis tissue. We first examined hematoxylin and eosin stained lung sections for immune cells within the alveolar interstitium, and we found more inflammatory cell aggregates in HPS pulmonary fibrosis explants than in IPF explants or IPF biopsies (Figures 3A,B). Automatic quantification of macrophages in entire lung tissue sections by means of our dedicated algorithms was carried out from CD68 immunostaining (Figure 3C). We found that the percentage of CD68 immunostaining in HPS lung explant tissue was significantly higher than that in IPF biopsies (*p* = 0.016) (Figure 2E). Although percentage of CD68 immunostaining in IPF explants was lower than that in HPS explants and higher than that in IPF biopsies, these differences were not statistically significant (*p* = 0.190 and *p* = 0.341, respectively).

### Ashcroft Scoring of Pulmonary Fibrosis

To determine whether our automated quantification of fibrosis is comparable to conventional methods to assess pulmonary fibrosis in human patient slides, Ashcroft scoring was performed. Intraclass correlation value of scores from three independent readers was 0.868. In contrast to automated quantitative analyses, mean Ashcroft score of HPS pulmonary fibrosis explant tissue did not differ significantly from that of IPF explants (Figure 2F). However, the mean Ashcroft score of IPF biopsy tissue was significantly lower than either HPS pulmonary fibrosis or IPF explants (*p* = 0.003 and *p* = 0.004, respectively), which was in agreement with results of the automated quantitative analyses.

### Correlation Between Automated Digital Quantification and Pulmonary Function Measurements

**Figure 4:**
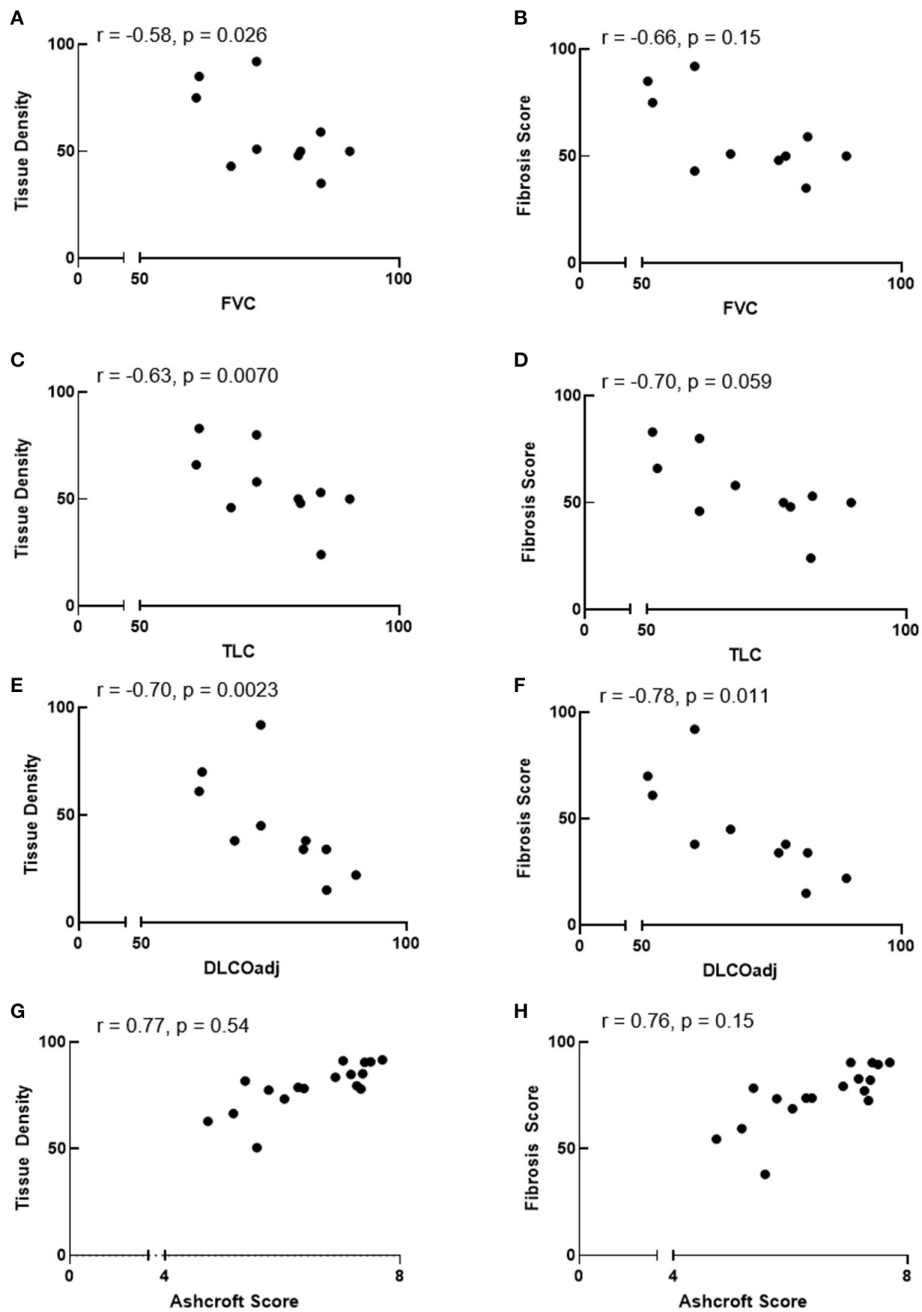
Correlation between automated quantification values and pulmonary function or ashcroft scores. **(A–F)** Automated tissue density correlate inversely with FVC and TLC and automated tissue density and fibrosis score inversely correlate with DLCOadj. **(G,H)** Comparisons between automated values and Ashcroft scores showed a trend between fibrosis scores and Ashcroft scores. r = Spearman correlation coefficient.

We sought to determine whether automated tissue density and fibrosis score values correlate with pulmonary function test measurements or Ashcroft scores. Linear regression analysis showed that automated tissue density inversely correlated with FVC and TLC (r = −0.58, *p* = 0.026 and r = −0.63, *p* = 0.007, respectively), and fibrosis score values tended to correlate inversely with FVC and TLC (r = –.66, *p* = 0.15 and r = −0.70, *p* = 0.059, respectively) (Figures 4A–D). Automated tissue density and fibrosis scores inversely correlated with DLCOadj (r = −0.70, *p* = 0.002 and r = −0.78, *p* = 0.011, respectively) (Figures 4E,F). Comparisons between automated measurements and Ashcroft scores showed a trend between automated fibrosis score values and Ashcroft scores (r = 0.76, *p* = 0.15, respectively) (Figures 4G,H).

## Discussion

We show that the automated digital quantification we developed in the present study for assessment of pulmonary fibrosis in human lung specimens from patients with HPS or IPF is advantageous because this method provides accurate, reliable and reader-independent continuous measurements of fibrosis severity in entire lung tissue sections. The potential value of the automated tissue density and fibrotic score as a direct read out of the severity of pulmonary fibrotic alterations was emphasized by their good correlation with pulmonary function measurements. It is noteworthy that in contrast to murine lung tissue, mucus was abundant in airspaces of human samples and was an obstacle to the selective digital analysis of fibrotic lung tissue. The development of combined coloration of picrosirius red with alcian blue allowed us to resolve this issue by selectively discriminating parenchymal tissue from airspace mucus. We also recognize that tissue density measurements may be affected by lung inflation, which should be considered in pre-clinical animal studies utilizing this method to automatically quantify fibrosis. In this study, however, none of the human lung explant specimens were inflation fixed.

We anticipated that automated quantification of tissue density and fibrosis scores would be higher in IPF explants than IPF biopsies; patients who receive lung transplants have severe, end-stage disease, whereas those who undergo lung biopsy generally have mild or moderate disease. Our results showing significant differences in both automated tissue density and fibrotic scores between these two groups are in agreement with assessments of their clinical severity of disease. Lung physiology tests and molecular analyses, such as measurements of tissue hydroxyproline levels, are conventionally used to estimate severity of pulmonary fibrosis. Our data demonstrate that the present automated digital imaging analysis may potentially complement lung function tests and molecular assays by estimating severity of disease based on analysis of tissue histology. Notably, this method allows several parameters in the same lung section to be concurrently measured, including parenchymal tissue density, fibrosis score, and collagen content. Further studies analyzing larger groups of patients with pulmonary fibrosis of varying severity of disease are indicated to determine whether this digital imaging analysis is able to detect mild differences in severity of pulmonary fibrosis. This is likely possible because we previously showed that the automated tissue density and fibrosis score allow precise quantification of very low levels of pulmonary fibrosis induced by bleomycin concentrations up to 5-fold lower than usual doses in this experimental murine model (29).

We found that automated digital fibrosis measurements, and not Ashcroft scores, of HPS pulmonary fibrosis explants were significantly higher than IPF explants. Despite a trend in correlation between these two analysis methods, these findings indicate that our digital imaging analysis is more sensitive than Ashcroft scoring for quantifying tissue fibrosis. Ashcroft scoring is a widely used method to quantify pulmonary fibrosis based on histological analysis. However, Ashcroft scoring has limitations, including variability between scorers, subjective assignment of scores, use of a discontinuous scale consisting of only nine integers ranging from 0 to 8, and analysis of several selected, but not all, fields from each tissue section. In contrast, our digital imaging analysis is automated and reader-independent and quantifies fibrosis using a continuous scale in entire tissue sections after automatically excluding large blood vessels and airways.

Results from our digital imaging analyses in this pilot study indicate that these HPS pulmonary fibrosis explants have more fibrosis than these IPF explants. Differences in age of onset of disease suggest that HPS pulmonary fibrosis is more aggressive, and thus perhaps more severe, than IPF. Pulmonary fibrosis generally manifests in middle aged adults with HPS-1 and HPS-4 as well as in children with HPS-2 (13, 40). In contrast, IPF typically affects older individuals (1). We explored whether altered accumulation of extracellular matrix protein or immune cells may contribute to the difference in automated digital imaging analyses between HPS pulmonary fibrosis and IPF. We determined that collagen content was similar in both disorders despite the difference in automated fibrotic score. One potential explanation is the presence of more immune cell aggregates in HPS pulmonary fibrosis than IPF tissues. Although pulmonary macrophage percentage was higher in HPS pulmonary fibrosis explants compared to IPF explants, this difference was not significant in our analysis using limited numbers of tissue sections. We acknowledge that it is possible that analyses with more specimens might detect differences in lung macrophage content between HPS pulmonary fibrosis and IPF explants. However, HPS is a rare disorder, and few patients with HPS pulmonary fibrosis have received lung transplants. Thus, the availability of specimens from patients with HPS pulmonary fibrosis is limited. In addition to potential differences in numbers of lung macrophages, dysfunction of these cells may also contribute to a profibrotic milieu in HPS, because alveolar macrophages are activated in HPS (39).

Quantitative digital image analysis methods of lung morphometry have been introduced, but a limitation is their requirement of manual interventions (21–28). Consequently, these techniques are laborious, time consuming, and subject to researcher intra- and inter-variabilities. Also, some available digital imaging analysis methods are performed using selected regions of interest or microscopic fields of view, and thus exclude several areas of lung sections. In the present study, we report that this digital imaging analysis is more sensitive than conventional Ashcroft scoring for measuring pulmonary fibrosis in human lung tissue samples. This digital imaging analysis is fully automated and reader-independent, and it allows concomitant quantification of multiple parameters of fibrosis on a single stained lung section. We also show that digital imaging analysis can provide automated quantification of immunolabeled cells in human lung tissue sections. This method can also be utilized to quantify fibrosis in other organs. Indeed, other reports show that tissue density and fibrosis in murine models of non-alcoholic fatty liver disease and non-alcoholic steatohepatitis were quantified using this digital imaging analysis (23, 41).

In conclusion, severity of pulmonary fibrosis in stained human tissue sections can be quantified using automated digital imaging analysis and offers important advantages compared to conventional semi-quantitative methods to assess lung fibrosis. Digital imaging analysis can also be utilized for automated quantification of immunostained cells in fibrotic lung tissue sections, which indicates its potential value in providing deep phenotyping and comprehensive quantification of inflammation in other diffuse lung diseases. This histological quantitative analysis may complement other conventional measurements of pulmonary fibrosis severity and has potential application in preclinical studies and clinical investigations focusing on fibrotic lung disease and other diffuse pulmonary diseases, such as sarcoidosis, hypersensitivity pneumonitis, or COVID-19.

## Data Availability Statement

The datasets presented in this article are not readily available because data may be made available upon written request to the corresponding author. Requests to access the datasets should be directed to Bernadette R. Gochuico, gochuicb@mail.nih.gov.

## Ethics Statement

The studies involving human participants were reviewed and approved by Institutional Review Board, National Human Genome Research Institute, NIH. The patients/participants provided their written informed consent to participate in this study.

## Author Contributions

LT, YJ, LL, KB, MM, KO'B, SN, DV, SE-C, MM, and BG contributed to the conception and design, or acquisition of data, or analysis and interpretation of data, drafting the manuscript or revising it critically for important intellectual content, and final approval of the version to be published. All authors contributed to the article and approved the submitted version.

## Conflict of Interest

YJ was a Co-founder, Co-owner, and Chief Scientific Officer of Biocellvia. KB is an employee of Biocellvia. A patent application related in part to this work was filed. The remaining authors declare that the research was conducted in the absence of any commercial or financial relationships that could be construed as a potential conflict of interest.
